# Association of Tetrahydrocannabinol Content and Price in Herbal Cannabis Products Offered by Dispensaries in California: A Purview of Consumers/Patients

**DOI:** 10.3389/fpubh.2022.893009

**Published:** 2022-06-17

**Authors:** MaryBeth Dobbins, Mannat Rakkar, Katharine Cunnane, Sarah D. Pennypacker, Kimberly G. Wagoner, Beth A. Reboussin, E. Alfonso Romero-Sandoval

**Affiliations:** ^1^Department of Anesthesiology, Pain Mechanisms Laboratory, Wake Forest University School of Medicine, Medical Center Boulevard, Winston-Salem, NC, United States; ^2^Department of Social Sciences and Health Policy, Wake Forest School of Medicine, Medical Center Boulevard, Winston-Salem, NC, United States; ^3^Department of Biostatistics and Data Science, Wake Forest School of Medicine, Medical Center Boulevard, Winston-Salem, NC, United States

**Keywords:** Delta-9 tetrahydrocannabinol, cannabidiol, drug policy, marijuana, medical marijuana, legalization, dispensaries, price

## Abstract

**Background and Aims:**

The U.S. legal cannabis market is saturated with products containing high levels of tetrahydrocannabinol (THC), with no distinction between medical and recreational programs. This omnipresence of potent cannabis products seems to be driven by the recreational realm, where cannabis with the highest THC content is prized. This prevalence of highly potent cannabis is conveyed to medical programs, which places consumers (patients) at higher risk for over consumption and cannabis use disorder. Thus, understanding what factors influence the market that patients face in medical cannabis programs could shed light on the risks of legal cannabis. The supply and demand dynamic of the US for-profit cannabis market could explain the current market composition; therefore, we postulate that a financial gain could influence the perpetuation of the prevalence of high THC products in legal cannabis dispensaries. We investigate whether THC content in popular cannabis products correlates with higher prices and assess whether some attributes (type of product, chemovars, or presence of cannabidiol (CBD) affect the association of THC with price.

**Methods:**

We focus on the world's largest cannabis market, California. We randomly selected dispensaries across the state, screened for a web presence and product menu, determined the most prevalent product type, and collected THC and CBD concentration, price, and other product attributes.

**Results:**

We observed that herbal products were more common, they had THC concentrations greater than 10%, and THC concentrations positively correlated with price. This correlation existed in flower and preroll presentations, all chemovar, and independently of the level of CBD. CBD did not correlate with price; however, the presence of CBD diminished the THC and price correlation particularly in products with high THC (>15%).

**Conclusions:**

Overall, highly potent herbal cannabis products (>15% THC) are the majority of products offered and more expensive regardless of product type or chemovar in California dispensaries, suggesting that a financial gain contributes to the current market composition. Efforts to limit the availability of highly potent THC products and educate consumers about potential harms are needed.

## Introduction

Delta-9-Tetrahydrocannabinol (THC), the primary active component of cannabis, is responsible for the psychotropic effects associated with cannabis, including its medicinal effects (1). Cannabidiol (CBD), one of the more prevalent active ingredients of cannabis possesses potent antiepileptic (2, 3), and potential anxiolytic effects (4). Cannabidiol could reduce or enhance the effects of THC (5).

The effects of THC and CBD are dose dependent and medicinal or intoxicating effects are achieved using different concentrations of THC and/or CBD. Yet, the market of available products offered from dispensaries fail to reflect accessible means for patients and participants to suitably implement appropriate dosing. Products with low levels of THC are most suitable for medicinal purposes. Cannabis with 2-10% THC (6–11) can reduce chronic pain and 5–10 mg p.o. has been shown to reduce nausea and increase appetite (12). However, highly potent cannabis products dominate both medical and recreational programs in the U.S. (13). Chemovars with high THC concentrations (>15%) are more commonly available from dispensaries as available chemovars were already increasing their THC concentrations when cannabis reform was implemented (14). While high THC cannabis produces strong psychotropic hedonic effects, they can also produce acute severe adverse effects (15–18). How the current medical cannabis programs ended offering highly potent and less suitable medicinal products is not completely understood. This study aims to investigate how a for-profit market dynamic could alter the type of products offered in cannabis programs in the U.S.A. and affect the medical options for patients seeking in cannabis the relief that cannot be found in modern medicine products.

In a for-profit business model, supply and demand control the market (19, 20). In fact, retailers have been postulated to be major drivers of potency in the available cannabis products (21), perhaps in response to consumers' demand (22). As expected, legal dispensaries seem to use this consumer preference for economic benefit. Indeed, as consumers' legal risks are reduced, prices of products rise in the short term (23). Hence, recreational or dual medical/recreational consumers display a higher willingness to pay for cannabis products with high THC content (24), as observed in Washington state between 2014 and 2017 (25). Consequently, it is possible that the current legal supply and demand dynamics explain the omnipresence of highly potent products in both medical and recreational programs, and that a financial gain enhances a feedback loop that perpetuate and enhance the current market composition.

One of the possible consequences of using a similar dynamic in both types of programs is medical insights into THC potency could be overlooked by factors impacting profits and related to recreational use. For this reason, we focus on a U.S. cannabis market that is composed of both medical and recreational programs. We chose the largest cannabis market in the world, California (26), to test the hypothesis that higher THC content in cannabis dispensary products is positively correlated with higher prices, and that this correlation is not altered by major product attributes. We focus on the online market because online advertising is a major marketing strategy for cannabis (27–29).

We tested our hypothesis following these specific aims: (1) determine what is the most prevalent type of cannabis products offered in the California market; (2) correlate the THC concentration of cannabis products with their retail price using the most prevalent types of products; (3) assess whether different chemovars (Sativa, Indica, Hybrid) alter the potential correlation, or lack thereof, between THC and price; and (4) evaluate whether the presence of CBD affects the potential correlation, or lack thereof, between THC with price. We focus on these aspects because price, chemovar type, THC content, and CBD amount ranked in the top five most important attributes of cannabis products considered by consumers, thus retailers may use these to influence pricing of products (22).

## Materials and Methods

### Dispensary Source

We utilized a business list from the California Bureau of Cannabis Control's (BCC) directory to identify dispensaries (30). This document was last updated to include all businesses with an active license approved by the BCC as of September 2018— there were 411 licensed retailers of medical or recreational cannabis listed. We accessed and collected businesses from this list between August 2019 to April 2020. We verified dispensaries by their registration for a license through the BCC, a physical address listed on an independent website, and verification of the business' address on Yelp.com (30–33). We collected data specifically accessible from their independent websites. We excluded dispensaries without (1) web presence or website, and (2) online presence only via third-party website like weedmaps.com, leafly.com, heartjane.com ortreez.io.

### Examination of Product Types Featured on Dispensary Websites

For this study (study 1), we randomly selected ten dispensaries from the BCC business list. We collected data on the first ten products displayed in the online menu since they occupy approximately 50–75% of the device screen (computer or cellphone), making them most salient to shoppers. We recorded the types of products (herbal [flower and preroll], vaping/cartridges, edibles, topical, concentrate, etc.) and their prevalence among the top ten products displayed. This provided the most common product categories featured in online dispensaries. We recorded THC and/or CBD content. If listed as a range (e.g., 30–40%), then the average of these values was used for analysis.

As secondary outcomes we also analyzed the rank of appearance of different types of products (flower, preroll, vaping/cartridges, edibles, etc.) in (1) the filter menu (i.e. drop down menu) that shows the product types or categories and requires a click to be displayed, and (2) the visual order of product categories that requires scrolling down the webpage, does not depend on the filter menu, and could be independent from the top featured products. The filter menu and visual order of product categories could be used by some consumers to find specific types of products, and the order of appearance could influence the decision-making process of shoppers. For this analysis, we ranked the position in which product categories or types appear; i.e., if it appears first, a value of one was given, if second, the value given was two, etc. Dispensaries were excluded if their product types were organized with no grouping of product types or alphabetically. Three dispensaries were excluded from filter menu analysis and one dispensary was excluded from visual order of product categories due to these reasons. Since online advertising changes frequently, we repeated the same menu and product data collection strategies on the same ten dispensaries approximately 5 weeks later to determine whether there was a difference in the menu formatting and featured products of each online dispensary.

### Correlation of THC Concentration and Price

Study two was based on results of study one and used slightly different inclusion criteria (Supplemental Table 1). We increased the number of dispensaries analyzed (~10% of dispensaries listed by the BCC in California in 2018) and collected information of all herbal products that met inclusion criteria (Supplemental Table 2). Consequently, we sampled 137 dispensaries, from which 41 met the listed criteria. Most of the businesses collected had licenses for dual designations, 13 were only medicinal, and one was only adult-use. The inclusion/exclusion criteria of product information collection are listed in Supplemental Table 2. The following product information was collected: name of product, type of product (flower or preroll), chemovar (if included; Sativa, Indica, Hybrid; if “Indica-dominant” or “Sativa-dominant” these were recorded under Hybrid), THC content (if listed as range, then the average of these values was recorded), CBD content (if included), price per weight in US$ (normalized to US$/1 gram). If a product had multiple amounts and multiple prices listed, only the price listed for 1 g was included.

### Statistical Analysis

Linear regression analyses were conducted to examine the association between THC content and price of products. Average price was also compared using *t*-tests (unpaired, one- or two-tailed where applicable), or one way-ANOVA + Tukey's posttest where appropriate, and a *P* < 0.05 was used as the level of significance.

## Results

### Product Type Prevalence

Most products featured in the top ten on online menus are herbal, 68% flower and preroll (62% and 6%, respectively), followed by 8% edibles, 8% topicals, 7% vape/cartridge, and 9% other forms (Supplemental Table 3). Flower products were featured within the top ten products by 80% of dispensaries while prerolls and other products were in 30% or less (Supplemental Table 1). Within the top ten products displayed online, we found that a median of 9.5, 3, and 3 were flower, preroll, and concentrate respectively (Supplemental Table 3). Herbal products were also prioritized on the filter menu (Supplemental Table 4) and in the visual order of product categories (Supplemental Table 5). These results are consistent with our previous analysis made approximately 5 weeks earlier (Supplemental Tables 3–5).

### THC Content in More Prevalent Product Types

From the 62 flower products displayed in the top ten of the dispensaries analyzed, only 35 had THC content information, and 18 had CBD content information. From the 6 preroll products displayed in the top ten of the dispensaries analyzed, four had THC content information and only one had CBD information. Both flower and preroll have high levels of THC (24.57 ± 4% and 20.22 ± 0.9%, respectively), and low levels of CBD (3.34 ± 9.3% and 1 ± 1%, respectively; Supplemental Table 6). Notably, none of the products prioritized (top ten displayed) online had <15% THC.

### THC Content and Price Correlation by Product Type

**Figure 1:**
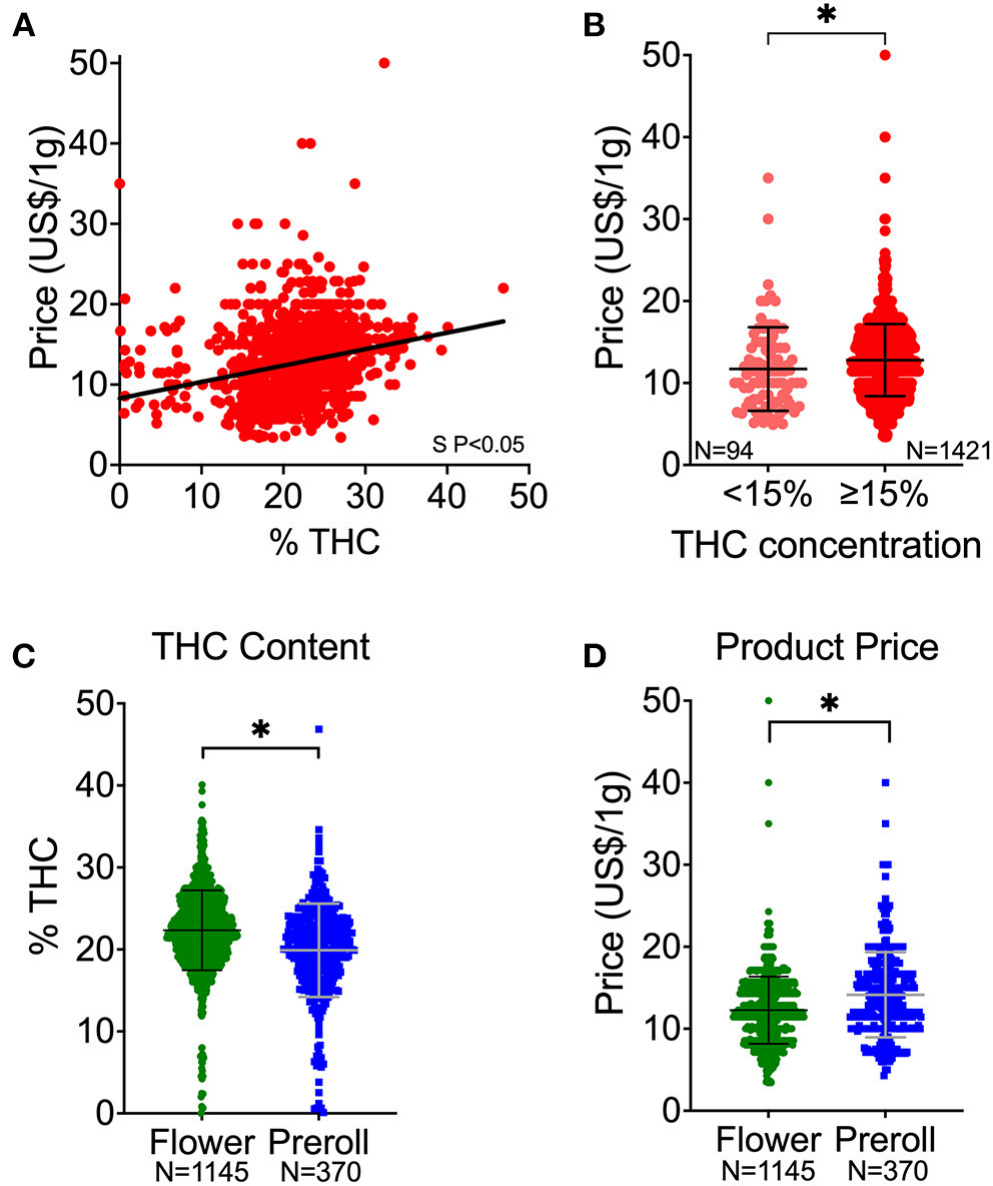
Cannabis potency (THC content) and price associations. **(A)** THC and price correlation of all herbal products. **(B)** Price distribution of products with <15% THC in comparison to products with ≥15% THC. **(C)** Comparison of THC content (%) between flower and preroll products. **(D)** Comparison of price (US $) between flower and preroll products. S P < 0.05 denote significantly non-zero slope **(A)**. *P < 0.05 between the mean values of the groups **(B–D)** by Welch's *t*-test (unpaired, two-tailed). Data shown as mean ± SD **(B–D)**.

Since study 1 revealed that herbal cannabis was the most frequent product, we subsequently focused on herbal (i.e., flower and preroll) cannabis to determine potential THC content and price correlation. Additionally, we observed in study one that herbal products consistently have high levels of THC, which prevents an accurate correlation of THC and price since there are few low THC cannabis products available. Thus, we increased the number of dispensaries to have a better representation of the available products. In total, our sample includes 1,515 herbal products. The products' THC content ranged from 0% to 47% and Price (US$/g) ranged from $3.49 to $50.00. We found price increased as products became more potent, as illustrated by a positive non-zero slope (Figure 1A). The average price of the high potency products (≥15% THC) is significantly greater than low potency products (<15% Figure 1B), confirming the positive correlation between THC content and price in herbal products.

**Figure 2:**
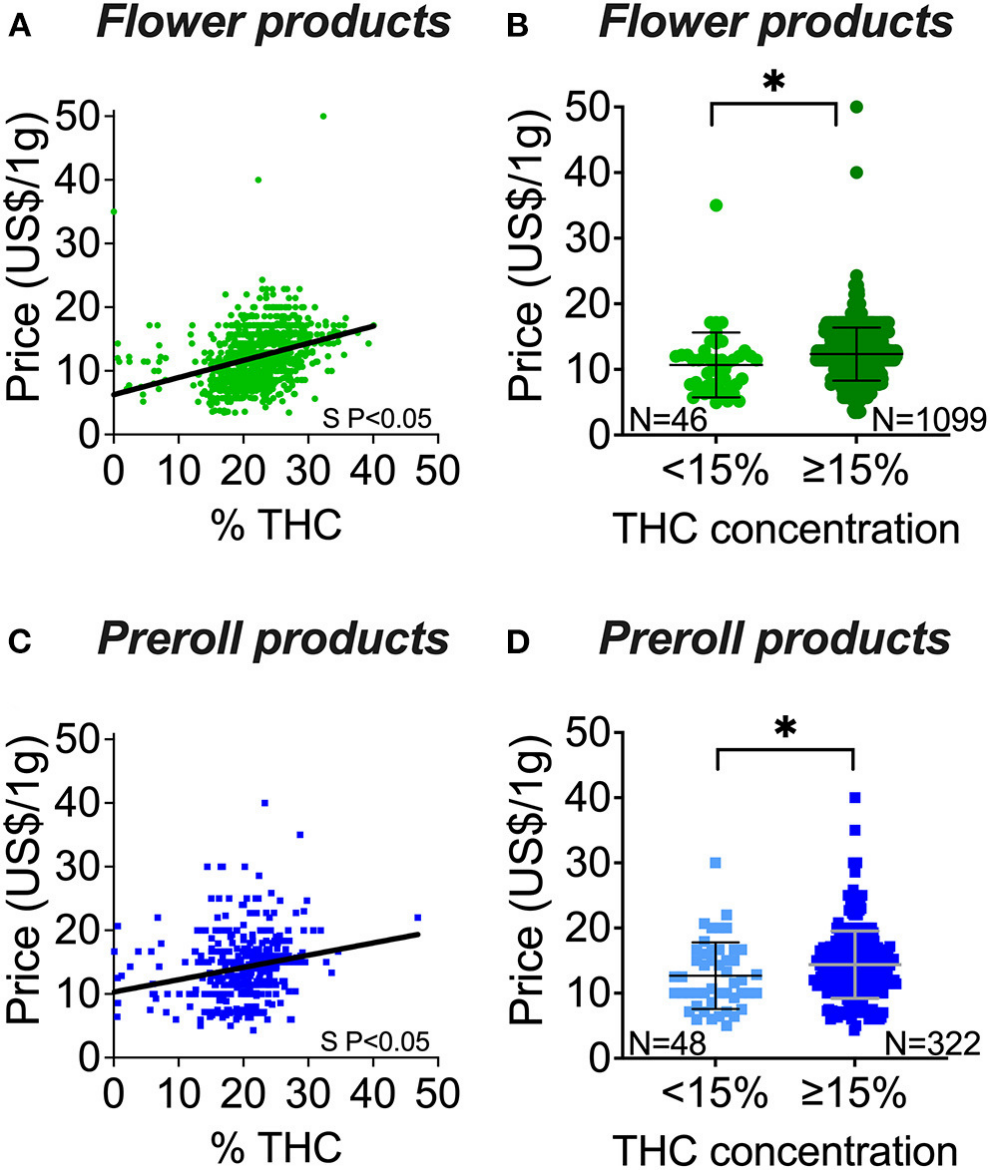
Flower and preroll cannabis potency (THC content) and price associations. **(A)** THC and price correlation of flower products. **(B)** Price distribution of flower products with <15% THC in comparison to flower products with ≥15% THC. **(C)** THC and price correlation of preroll products. **(D)** Price distribution of preroll products with <15% THC in comparison to preroll products with ≥15% THC. S P < 0.05 denotes significantly non-zero slope **(A,C)**. *P < 0.05 between the mean values of the groups **(B,D)** by Welch's *t*-test (unpaired, two-tailed). Data shown as mean ± SD **(B,D)**.

Between the types of products, flower had higher potency (THC content) than preroll (Figure 1C). However, prerolls are generally more expensive than flower (Figure 1D). We observed that THC content was positively correlated to price and that the price of the high potency products (>15% THC) is significantly greater than in low potency products (<15% THC) in both flower and prerolls (Figure 2).

### THC Content and Price Correlation by Chemovar

**Figure 3:**
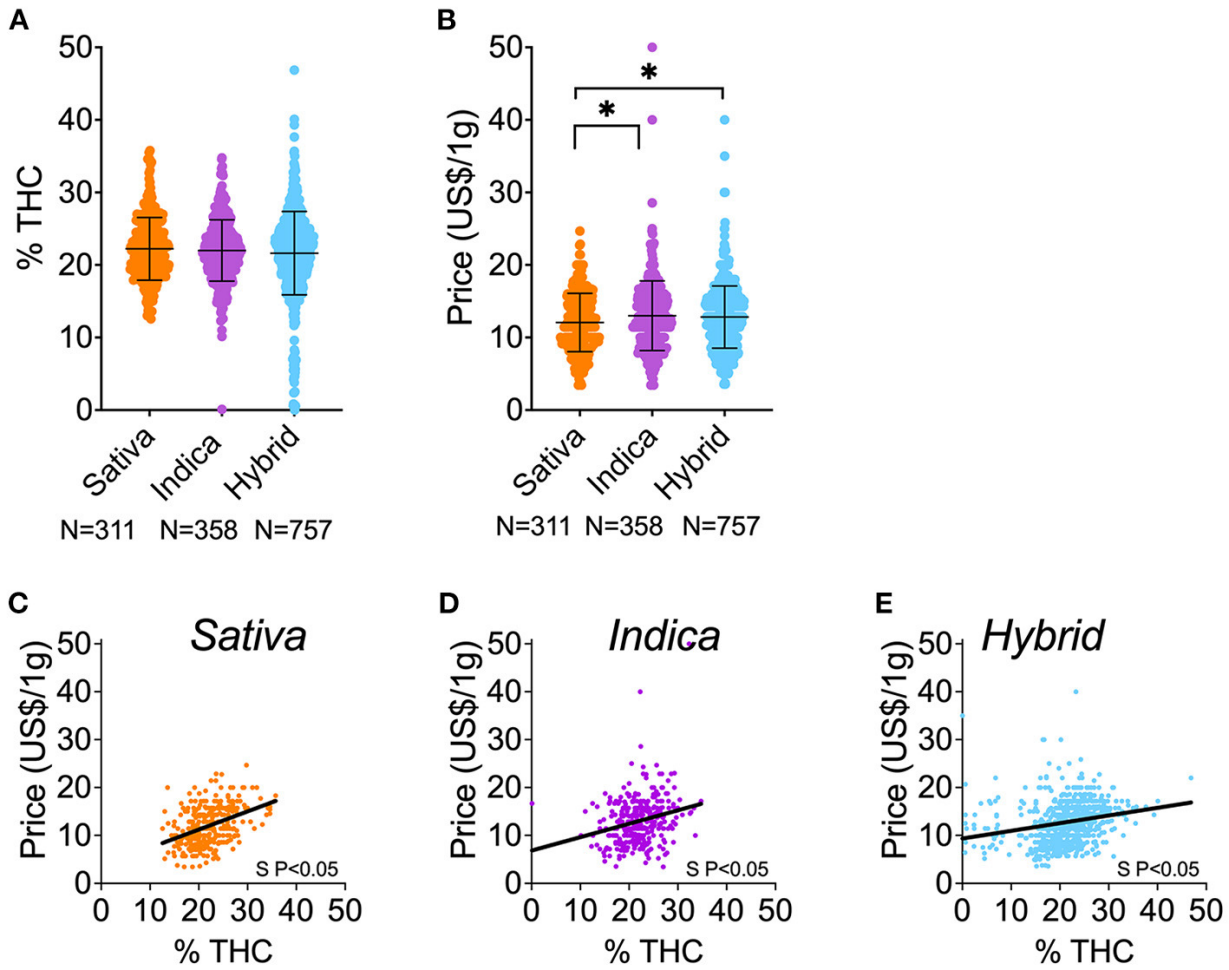
THC content and price of cannabis herbal products by chemovar. **(A)** Comparison of THC content (%) among Sativa, Indica, and Hybrid herbal products (flower and preroll). **(D)** Comparison of price (US $) among Sativa, Indica, and Hybrid herbal products (flower and preroll). **(A)** THC and price correlation of Sativa herbal products. **(B)** THC and price correlation of Indica herbal products. **(C)** THC and price correlation of Hybrid herbal products. *P < 0.05, between linked groups by one-way ANOVA, Tukey's posttest **(A,B)**. Data shown as mean ± SD **(A,B)**. S P < 0.05 denotes significantly non-zero slopes **(C–E)**.

First, we observed that products identified as Indica, Sativa, or Hybrid were similar in potency (above 20% THC for the three groups), but Hybrid products were two times more abundant and displayed a wider range of THC content than Sativa and Indica (Figure 3A). These similarities and pattern of THC content were observed in flower or prerolls independently (data not shown). Second, we observed that Indica and Hybrid products were significantly more expensive than Sativa products (Figure 3B), but this significance did not persist when we segregated the data in flower and preroll by individual chemovars (data not shown). Third, we found price increased as products became more potent (Figures 3C–E). In the Hybrid chemovar products (the most abundant), we observed that the ≥15% THC population has a higher price than in the <15% THC population, and this is also observed in flower or prerolls individually (Supplemental Figure 1). Since <15% THC products were rare in Sativa and Indica products, we could not compare the average price of this population with the price of >15% THC products.

### Association of CBD Content With Price

**Figure 4:**
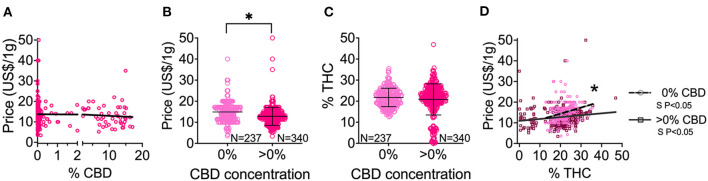
Cannabis CBD content (absence 0% or presence >0%) and price and THC associations in products with CBD information. **(A)** CBD and price correlation in herbal products. **(B)** Price distribution of products with 0% CBD in comparison to products with >0% CBD. **(C)** THC content distribution of all herbal products with 0% CBD in comparison to products with >0% CBD. **(D)** THC and price correlation comparing products with 0% CBD to products with >0% CBD. *P < 0.05, between groups by Welch's *t*-test (unpaired, two-tailed). Data shown as mean ± SD **(B,C)**. S *P* < 0.05 denotes significantly non-zero slope **(D)**; *P < 0.05 between group slopes **(D)**.

We observed no correlation between CBD content and price (slope not significantly different from zero; Figure 4A). Then, we conducted comparative analyses in products with no CBD content (0%) against products with any CBD content (>0%). First, we observed that products with 0% CBD are more expensive than products with >0% CBD (Figure 4B). This association remained when we compared flower or preroll products independently (Supplemental Figures 2A,B). Second, we found no difference in the THC content of products with 0% and >0% CBD (Figure 4C). However, the population of 0% CBD does not have products with lower levels of THC (<10% THC), and the population with >0% CBD displays a broad range of THC content, including very low levels of THC products (ranged from 0.1% THC to 47% THC; Figure 4C).

### Effects of CBD Content on THC-Price Correlation

We further investigate whether CBD affects the positive correlation of price and THC content. We found that the product price positively correlates with THC content in both 0% CBD and >0% CBD products (Figure 4D). However, the slopes between these groups were significantly different, showing that the correlation of price and THC content is more prominent in 0% CBD products (Figure 4D). We found that products with <15% THC and 0% CBD are very rare, preventing us from comparing them with other populations. Interestingly, we found that highly potent products (≥15% THC) with 0% CBD ($15.04 ± 4.59, *n* = 227) were consistently more expensive than counterpart products (≥15% THC) with >0% CBD ($12.83 ± 4.10, *n* = 294; Supplemental Table 7). This is also true when we compared flower or preroll independently (Supplemental Table 7).

## Discussion

The major finding of our study is that THC content in herbal cannabis products, the most common type, offered online in California dispensaries is positively correlated with higher prices. This correlation occurs regardless of the type of product, chemovar, and presence of CBD. Interestingly, increasing CBD content alone did not correlate with higher prices but the presence of CBD altered the THC/price correlation. Our thorough analysis unveils multiple nuances that help better understand the economic dynamics of the legal cannabis market in California that could explain its composition. These results can help create strategies to provide a safer and more suitable marketplace for patients that find relief in cannabis.

By studying herbal product attributes from the online user interface of dispensaries, we uniquely approached the cannabis market from the purview of the consumer. Our data seems to tightly reflect what is in physical stores and the content of these goods as our results closely align with highly accurate sales data in other U.S. states (20, 25, 34). Accordingly, herbal products are the most common cannabis form preferred, purchased, and used by both recreational and medical consumers in the U.S. and other countries (24, 25, 35–37). Even though some studies suggest that consumers might pay slightly different prices to those listed online (38), more robust data indicate that cannabis sales are more frequent for products containing high THC levels (21, 25). Together, the data suggests that the market strategically associates with consumers' preferences and uses online advertising to influence and reinforce consumers' behavior. The promotion of highly potent products could convey the idea that high THC concentrations are better for medicinal purposes and may warrant FDA intervention against the dissemination of misinformation (39). We do recognize though that the label cannabinoid concentrations listed in commercially available products might not represent accurately their actual concentration (40–44). Regardless of this likely discrepancy, our study focused on the content that consumers see when examining online cannabis products.

Intriguingly, we show that the dynamic of supply and demand for highly potent products observed in recreational cannabis markets (20, 24, 25) is also present in the historically medical cannabis market of California. This dynamic could influence the preference of products with high THC content, which is in accordance with the sales of higher potency products being at a premium over other less potent products (20, 25). This functional overlap in marketing and financial practices is an additional concern for patients who seek medicinal benefits from cannabis in programs where medicinal and recreational systems coexist, and where more medically suitable products (i.e., low THC) are virtually non-existent or scarce. Thus, the current cannabis market composition represents an additional barrier for patients that seek a medical benefit from cannabis to access safer treatment options. Interestingly, many medical cannabis users in California consumed cannabis daily when recreational adult use was not legal (45), and perhaps these consumers also influence the current offer of potent products. However, new medical users should have access to less potent products that are more medically suitable for them (i.e., for pain treatment). The risk derived from the consumption of potent cannabis is not less for recreational consumers [i.e., cannabis users do not titrate their dose intake when using more potent cannabis (46), users of higher potency products experience more side effects (47), and emergency visits have increased after legalization of recreational cannabis in the U.S. (48, 49)]; therefore new policies should also include this segment of the population.

The price of cannabis products is determined by multiple cultivation factors such as insect pest/disease prevention or control, increasing yield, and achieving desired terpene or cannabinoid content (50). Similarly, operational factors such as competition, compliance with local and or state regulations, and finance management are the top three business-related challenges that affect cost in the cannabis industry (50). However, other less tangible factors could influence the price of cannabis products. For example, consumers are directly influenced by factual or alleged attributes of cannabis products (19, 20, 23, 24). Retailers could therefore use these claims to promote their available products and to increase prices. Some attributes include the alleged effects of different chemovars, Indica vs. Sativa (51, 52). Hybrid chemovars allegedly produce different levels of effects found with Indica and Sativa (53, 54). Perhaps this is the reason Hybrid product prevalence is increased when recreational use is also present within a medical cannabis market (55). Accordingly, medical cannabis consumers prefer Hybrids, followed by Indica (preferred by chronic pain patients), and Sativa chemovars (53–55). This consumers' bias for Hybrid and Indica chemovars may imply a higher demand, thus this could explain the higher prices of these chemovars over Sativa products uncovered in our study. Also, this preference could explain the higher instance of cannabis use disorders observed in Hybrid consumers than in those who preferred Indica (53). The public health interpretation of our data is that the preferential use of Hybrids (the most prevalent and expensive chemovar) could create a positive perverse feedback loop that gives and reinforces the commercial power of the market to shape its composition for their financial benefit, i.e., consumers will be willing to pay their prices as they develop increased tolerance to cannabis, and potentially a cannabis use disorder. Accordingly, it has been shown that medicinal cannabis consumers show patterns of heavy use [use on a daily basis (56)]. It is possible that these intense users (medical and recreational) are the primary consumers from dispensaries, as observed in other geographical areas (57). This heavy cannabis use could provide higher tolerance to high THC levels, and thus it could influence the offer of potent products.

We found that CBD reduces the price of highly potent products. Interestingly, medical cannabis consumers are more attracted to CBD than to THC content (24). However, very few products chosen for medical use contain CBD (54) and most products containing CBD also contain ≥15% THC, which contradicts the low THC/high CBD preference of medical consumers (58). This marketing dynamic suggests that medical cannabis consumers are more likely exposed to risky cannabis products (high THC) that are economically more accessible.

A limitation of our study is that we collected data only in California. Nonetheless, the California cannabis market has great influence in other regions, as reflected in the composition of the cannabis market (13) or the THC and price of herbal products (25) in California and other U.S. states. Similar research is necessary for non-herbal products and for other types of markets where herbal products are not legally available (i.e., New York, U.S.). Similarly, more studies are needed for CBD in markets where THC is not legal.

## Conclusions

In conclusion, the for-profit nature of the cannabis market has perpetuated the dominance of risky products. New policies are required to reconcile the profit-driven nature of the U.S. cannabis market and increase availability of safer products. Removing the financial gain from the equation seems a difficult task based on taxation, production, and quality control costs (59). Nevertheless, separating recreational practices, preferences, and market composition from medical programs should be part of regulatory policies. High potency cannabis products in the medical realm are not scientifically justified (except for patients with cannabis tolerance) (1), therefore removing false claims in the cannabis programs should include the removal of potent cannabis for most medicinal purposes.

## Data Availability Statement

The raw data supporting the conclusions of this article will be made available by the authors, without undue reservation upon request.

## Author Contributions

Conceptualization, methodology, project administration, resources, and supervision: ER-S. Data curation, investigation, and writing—review and editing: MD, MR, KC, SP, KW, BR, and ER-S. Formal analysis: MD, KW, BR, and ER-S. Funding acquisition: ER-S, BR, and KW. Writing—original draft: MD and ER-S. All authors contributed to the article and approved the submitted version.

## Funding

Funding provided by the Department of Anesthesiology and Pilot Research Award by the Center for Addiction Research, Wake Forest University School of Medicine (ER-S), and National Institute of Health, NIDA grants R01DA053209 (BR) and R01DA051542 (KW).

## Conflict of Interest

The authors declare that the research was conducted in the absence of any commercial or financial relationships that could be construed as a potential conflict of interest.

## Publisher's Note

All claims expressed in this article are solely those of the authors and do not necessarily represent those of their affiliated organizations, or those of the publisher, the editors and the reviewers. Any product that may be evaluated in this article, or claim that may be made by its manufacturer, is not guaranteed or endorsed by the publisher.
