# Supplementary material for: Association of Tetrahydrocannabinol Content and Price in Herbal Cannabis Products Offered by Dispensaries in California: A Purview of Consumers/Patients
Source: Front Public Health. 2022 Jun 17;10:893009. doi: 10.3389/fpubh.2022.893009 (PMC9247604; doi:10.3389/fpubh.2022.893009)
Supplement: Supplementary file 1 [file Data_Sheet_1.pdf]

SUPPLEMENTAL FIGURES

Supplemental Figure 1. Price comparison of Hybrid herbal products with <15% THC and ≥15% THC. (**A**) Price and potency distribution of all Hybrid herbal products. (**B**) Price and potency distribution of flower Hybrid herbal products. (**C**) Price and potency distribution of preroll Hybrid herbal products. ***P<0.05 between groups by Welch's t-test (unpaired, one-tailed). Data shown as mean ± SD.

Supplemental Figure 2. Cannabis CBD content (absence 0% or presence >0%) and price associations in flower and preroll products with CBD information. (**A**) Price distribution and CBD content of flower products. (**B**) Price distribution and CBD content of preroll products. ***P<0.05, between groups by Welch's t-test (unpaired, two-tailed). Data shown as mean ± SD.

SUPPLEMENTAL TABLES

Supplemental Table 1. Inclusion and Exclusion criteria of Dispensary sampling and collection.

	Inclusion Criteria	Exclusion Criteria
Web Presence	◦ Independent Website Presence	◦ No web presence or website<br>◦ Presence only via third-party website like weedmaps.com, leafly.com, heartjane.com or treez.io
Menu	◦ Had a menu on their independent website<br>◦ Embedded menu from a third-party website on their independent website<br>◦ Link or access to a menu on the dispensary's page or on a third-party menu website	◦ No menu available or accessible from dispensary website<br>◦ No THC content available for any products
THC content of products	◦ Included at least one flower product with THC content. THC content listed in percentage form	◦ No flower products listed a THC content anywhere in the product description page<br>◦ THC amounts only in weight (mg) form on menu

Supplemental Table 2. Inclusion and Exclusion criteria of product collection from online menus of dispensaries.

Inclusion Criteria	Exclusion Criteria
◦ Herbal Products<br>◦ flower or preroll	◦ Not an herbal product (edibles, extract, oils, vape, tincture, topical)
◦ THC amount was available	◦ No THC content listed
◦ Percentage Form (2019)	◦ THC listed as gram amount (mg) – this format does not comply with CA regulations (2019)
◦ THC content located in product title, product specs, or paragraph description	◦ If THC content only available in product image
◦ Price listed<br>◦ Natural cannabinoids	◦ No price listed for the product<br>◦ Stated it was infused. If products had THC >50% we considered these products as potentially infused and they were excluded.
◦ Weight of the amount of product listed in product specs, paragraph description, or in image (grams) (converted to grams if listed as ounces)	◦ Weight could not be determined

Supplemental Table 3. Number and percent of product type in top 10 products displayed in online menus and percent of dispensaries displaying these products in top ten products.

Product Types in Top 10	Median (Range)	Average ± SD	% of Dispensaries that have Product in Top 10	% Total Product
Most Recent Analysis				
**Flower**	**9.5 (2-10)**	**7.8 (3.059)**	**80**	**62**
**Preroll**	**3 (2-4)**	**3 (1.414)**	**20**	**6**
Concentrate	3 (3)	3 (0)	10	3
Pill/Capsule	3 (3)	3 (0)	10	3
Edible	2 (1-5)	3 (2.082)	30	8
Topical	1 (1-6)	3 (2.887)	30	8
Vape/Cartridge	1 (1-5)	2 (2.309)	30	7
Beverage	1 (1)	1 (0)	10	1
Tincture	1 (1)	1 (0)	10	1
Analysis conducted five weeks earlier				
**Flower**	**9 (5-10)**	**8 ± 2.268**	**80**	**64**
Vape/Cartridge	5 (1-6)	4 ± 2.646	30	12
**Preroll**	**2.5 (1-4)**	**2.5 ± 1.732**	**40**	**10**
Edible	2.5 (1-4)	2.5 ± 2.121	20	5
Pill/Capsule	3	3	10	3
Other/Misc	2	2	10	2
Beverage	2	2	10	2
Concentrate	1	1	10	1
Topical	1	1	10	1

Supplemental Table 4. Rank of product types found in filter menus and percent of dispensaries displaying these types of products in their filter menus.

**Menu Product Type Filters**	**Median (Range)**	**Average ± SD**	**% of Dispensaries that have product in filter menu**
Most Recent Analysis			
**Flower**	**1 (1-4)**	**1.7 ± 1.3**	**100**
Vape/ Cartridge	2 (2-3)	2.2 ± 0.5	100
Edible	3 (1-5)	3.3 ± 1.8	100
**Preroll**	**3 (2-6)**	**3.6 1.8**	**71**
Concentrate	4 (3-4)	3.8 ± 0.5	57
Extract	4	4	43
Sublingual	6	6	14
Other/Misc	6.5 (5-8)	6.5 ± 1.4	86
Topical	7 (5-7)	6.4 ± 0.8	100
Plant/Seeds	7.5 (6-9)	7.5 ± 2.1	29
Beverage	8.5 (8-9)	8.5 ± 0.7	29
Tincture	9 (6-11)	8.8 ± 2.2	57
Pill/Capsule	9.5 (9-10)	9.5 ± 0.7	29
Analysis conducted five weeks earlier			
**Flower**	**1 (1-5)**	**2.3 ± 1.704**	**100**
Vape/Cartridge	2 (1-3)	2 ± 0.816	100
Edible	3 (1-5)	3 ± 1.528	100
**Preroll**	**3 (2-6)**	**3.6 ± 1.817**	**71**
Concentrate	4 (3-4)	3.75 ± 0.5	57
Extract	4	4 ± 0.0	43
Sublingual	6	6.0	14
Other/Misc	6.5 (5-8)	6.5 ± 1.378	86
Topical	7 (5-7)	6.4 ± 0.787	100
Plant/Seeds	7.5 (6-9)	7.5 ± 2.121	29
Beverage	8.5 (8-9)	8.5 ± 0.707	29
Tincture	9 (6-11)	8.8 ± 2.217	57
Pill/Capsule	9.5 (9-10)	9.5 ± 0.707	29

Supplemental Table 5. Rank of product type found in the visual scroll down order of product category and percent of dispensaries displaying these types of products in in the scroll down order of product category.

**Product Categories**	**Median (Range)**	**Average ± SD**	**% of Dispensaries that have Product in scroll down categories**
Most Recent Analysis			
**Flower**	**1**	**1 ± 0**	**100**
Vape/ Cartridge	2 (2-4)	2.5 ± 0.8	89
Edible	3 (2-5)	3.3 ± 1	100
Concentrate	4 (2-5)	3.5 ± 1.2	67
Extract	4	4 ± 0	33
**Preroll**	**5 (2-6)**	**4.3 ± 1.4**	**78**
Topical	6 (4-7)	6.1 ± 1	89
Sublingual	6	6.0	11
Plant/Seeds	8 (6-9)	7.5 ± 2.1	22
Other/Misc	7 (5-8)	6.6 ± 1.1	78
Beverage	8.5 (8-9)	8.5 ± 0.7	22
Pill/Capsule	9.5 (9-10)	9.5 ± 0.7	22
Tincture	10 (8-11)	9.7 ± 1.5	33
Analysis conducted five weeks earlier			
**Flower**	**1**	**1 ± 0.00**	**89**
Vape/Cartridge	2 (1-4)	2.4 ± 0.916	89
Edible	3 (2-5)	3 ± 0.866	100
Concentrate	4 (2-5)	3.5 ± 1.225	67
Extract	4 (4-5)	4.3 ± 0.577	33
**Preroll**	**5 (2-6)**	**4.3 ± 1.380**	**78**
Topical	6 (4-7)	5.9 ± 1.246	89
Sublingual	6	6	11
Plant/Seeds	6	6	11
Other/Misc	7 (5-8)	6.6 ± 1.134	78
Beverage	8.5 (8-9)	8.5 ± 0.707	22
Pill/Capsule	9.5 (9-10)	9.5 ± 0.707	22
Tincture	10 (6-11)	9 ± 2.646	33

Supplemental Table 6. THC and CBD concentration from top ten products surveyed that included this information.

	Flower (%)		Vape/Cartridge (%)		Preroll (%)		Edible (mg/product)		Beverage (mg/serving)		Topical (mg/application or ounce)		Pill/Capsule (mg/capsule)	
	THC	CBD	THC	CBD	THC	CBD	THC	CBD	THC	CBD	THC	CBD	THC	CBD
**Average**	24.57	3.34	63.41	20.50	20.23	1	63.13	73	10	5	82.5	45	62.5	N.I.
**Median**	24.7	0.1	70.6	20.5	20.45	1	75	95	10	5	60	10	62.5	
**SD**	4.01	9.31	22.09	27.57	0.93		42.00	48.17	0	0	45	70	53.03	
**N**	35	18	3	2	4	1	8	5	2	2	4	4	2	0

N.I., no information provided

Supplemental Table 7. Price comparison between products with 0% or >0% CBD in products with ≥15% THC in all herbal products or divided in flower and preroll products.

Price (US$/1g)	≥15% THC		
	**0% CBD**	**>0% CBD**	**P-value**
**All Products**	15.04±4.59 n=227	12.83±4.10 n=294	*
**Flower**	14.41±3.87 n=113	12.77±4.09 n=253	*
**Preroll**	15.66±5.15 n=114	13.24±4.24 n=41	*

*****P<0.05, between 0% and >0% CBD groups by Welch's t-test (unpaired, two-tailed).
