# Supplementary figures and images for: Association of Tetrahydrocannabinol Content and Price in Herbal Cannabis Products Offered by Dispensaries in California: A Purview of Consumers/Patients
Source: Front Public Health. 2022 Jun 17;10:893009. doi: 10.3389/fpubh.2022.893009 (PMC9247604; doi:10.3389/fpubh.2022.893009)

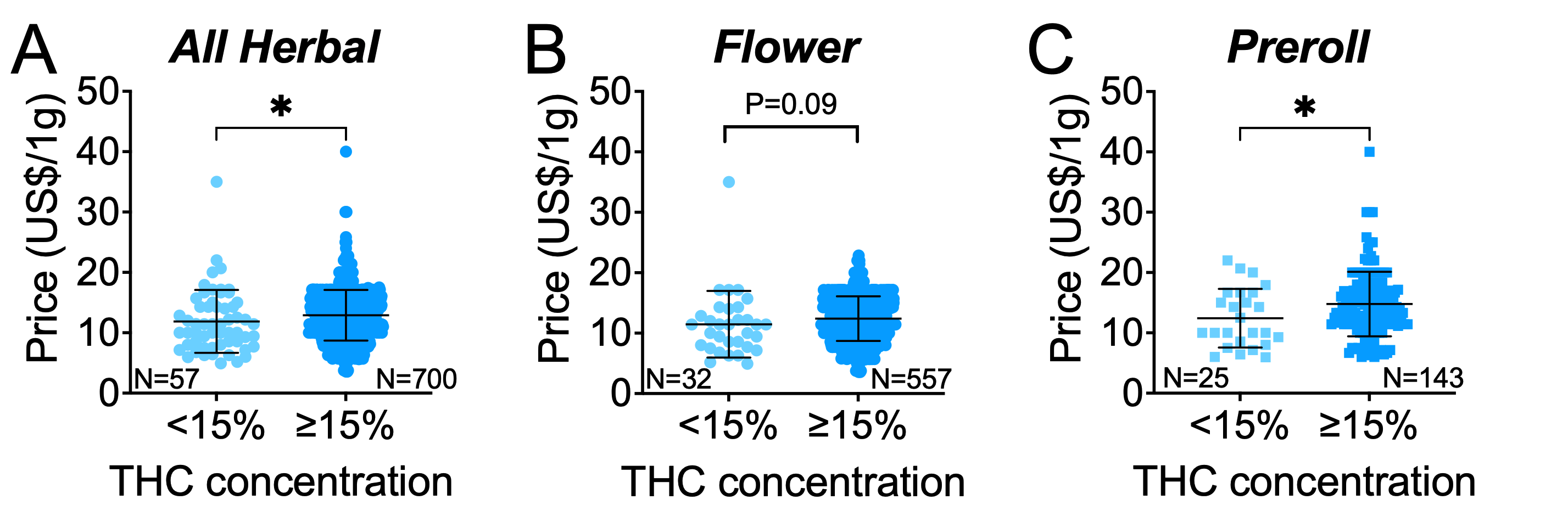

Supplement: Supplementary file 2 [file Image_1.TIFF]

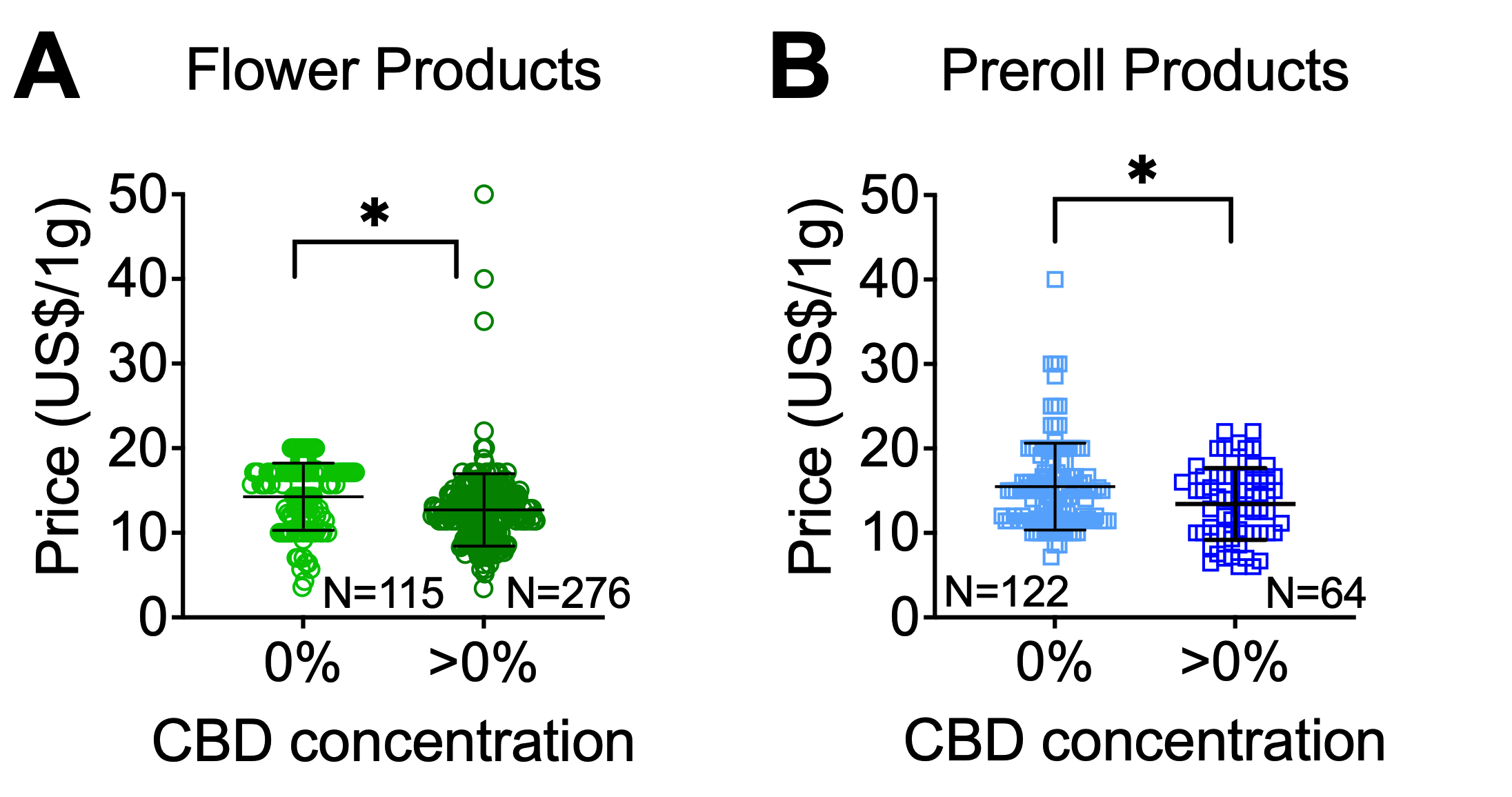

Supplement: Supplementary file 3 [file Image_2.TIFF]
